# Case Report: Sequential mNGS captures fatal HSV-1 reactivation after intestinal obstruction-associated ARDS in a 91-year-old

**DOI:** 10.3389/fmed.2026.1910309

**Published:** 2026-09-14

**Authors:** Jiahuan Li, Xiaofei Li, Huafang Zhong, Li Wang, Ling Yue

**Affiliations:** Department of Cadre Ward Two, Central Theater Command General Hospital of the PLA, Wuhan, China

**Keywords:** ARDS, case report, elderly, HSV-1, intestinal obstruction, mNGS

## Abstract

Herpes simplex virus type 1 (HSV-1) is a ubiquitous pathogen. Nevertheless, disseminated HSV-1 infection with confirmed viremia and fatal acute respiratory distress syndrome (ARDS) following intestinal obstruction in very old individuals is exceedingly rare. Here we report a 91-year-old patient with previously intact immunity who developed ARDS after incomplete intestinal obstruction, followed by HSV-1 reactivation. Although routine microbiological tests remained negative, five consecutive metagenomic next-generation sequencing (mNGS) assays dynamically tracked the evolving pathogens throughout the disease course. Despite broad-spectrum antibiotics, antifungals, antivirals, corticosteroids, IVIG, and mechanical ventilation, the patient’s refractory hypoxemia remained uncorrected, leading to multiple organ dysfunction and death. This case highlights a rare clinical scenario and provides valuable insights for the diagnosis and management of critically ill elderly patients, offering a comprehensive, longitudinal perspective on the clinical application of mNGS.

## Introduction

Acute respiratory distress syndrome (ARDS) is a life-threatening condition with diverse etiologies, but its exact pathogenesis remains not fully understood. Previous research has indicated that there is an interaction and association between the gut microbiota and ARDS via the gut-lung axis (1–4). Herpes simplex virus type 1 (HSV-1) is a ubiquitous pathogen that establishes lifelong latency after primary infection. Reactivation is commonly triggered by immunosuppression or acute stress and typically presents as orolabial lesions. Despite high detection rates in the intensive care unit (ICU), the clinical relevance of pulmonary herpesviruses in ARDS remains controversial (5). In severely immunocompromised patients, particularly allogeneic stem cell and solid organ transplant recipients, HSV-1 pneumonia is rare but well-established and potentially life-threatening respiratory tract infections. In critically ill patients, their exact role in pulmonary injury is less clear. A clear diagnosis of HSV-1 pneumonia is difficult to establish because clinical criteria, radiological features, and laboratory findings all lack specificity. Current diagnosis relies on PCR or histopathology (6), but these methods lack sensitivity for dynamic monitoring. Recently, metagenomic next-generation sequencing (mNGS) has emerged as a powerful tool for identifying unexpected pathogens in critically ill patients (7–9), and offers new possibilities for early diagnosis and real-time pathogen tracking (10). Nevertheless, interpreting mNGS results remains challenging: a single negative assay does not exclude infection, detection of a microorganism does not prove causality, and the optimal specimen type and sampling timing are still debated (11).

This case presents a complete record of the entire disease course, with serial mNGS analyses performed on five consecutive occasions delineating the evolving pathogen profile throughout hospitalization (Table 1). This unique dataset provides rare, real-time insight into the gut-lung axis and the dynamics of viral reactivation in an aging host, highlighting the diagnostic value of serial mNGS on multiple specimens.

**TABLE 1:** Sequential metagenomic next-generation sequencing (mNGS) results.

Assay	Specimen	Day	Key findings (reads, relative abundance)	Interpretation
1	Sputum	12	EBV (29 DNA, 87.9%); oral commensals	Functionally negative
2	BALF	13	CMV (8 DNA, 0 RNA); *M. salivarium* (355 RNA, background)	Negative (latent CMV, no active replication)
3	BALF	21	HSV-1: 10,446 DNA/13,120 RNA (99.6%); *Candida parapsilosis* (high RNA); *Aspergillus flavus* (moderate)	HSV-1 pneumonia (fungi likely colonizers)
4	Blood	29	HSV-1: 469 DNA, 12,949 RNA (96.5%); CMV, EBV, TTV (low reads)	HSV-1 viremia (disseminated infection)
5	Lung tissue	Postmortem	HPyV7 (36 DNA); *Moraxella osloensis* (background)	Incidental, non-pathogenic detection

BALF, bronchoalveolar lavage fluid; EBV, Epstein–Barr virus; CMV, cytomegalovirus; *M. salivarium, Mycoplasma salivarium;* HSV-1, herpes simplex virus type 1; HPyV7, human polyomavirus 7; TTV, torque teno virus.

## Case presentation

On 26 February 2024 (day 1), a 91-year-old male patient underwent computed tomography (CT) due to a 3-day history of constipation and periumbilical pain. The clinical timeline, including all major clinical events, therapeutic interventions, laboratory parameters, body temperature, oxygenation index, and mNGS sampling points, is summarized in Figure 1. He had a history of coronary heart disease, hypertension, and persistent atrial fibrillation, with no known family history of genetic disorders, cardiovascular diseases, or malignancies. He had been living at home with the assistance of a caregiver prior to admission and was partially dependent on daily activities. He was on long-term oral antihypertensive and other medications for chronic conditions, with his blood pressure and heart rate well-controlled. Abdominal CT suggested incomplete intestinal obstruction (Figure 2A). After conservative management (fasting, enemas, gastrointestinal decompression, and cefoperazone-sulbactam), his abdominal pain gradually improved. On Day 7, the patient developed fever and cough. Repeat abdominal CT showed that the intestinal obstruction was relieved, but chest CT revealed patchy opacities in bilateral lungs (Figures 2B,C). Laboratory tests showed CRP 151.1 mg/L, IL-6 423.6 pg/mL, WBC 12.0 × 10^9^/L, neutrophils 90.6%, and lymphocytes 0.72 × 10^9^/L. Antibiotics were switched to meropenem.

**FIGURE 1:**
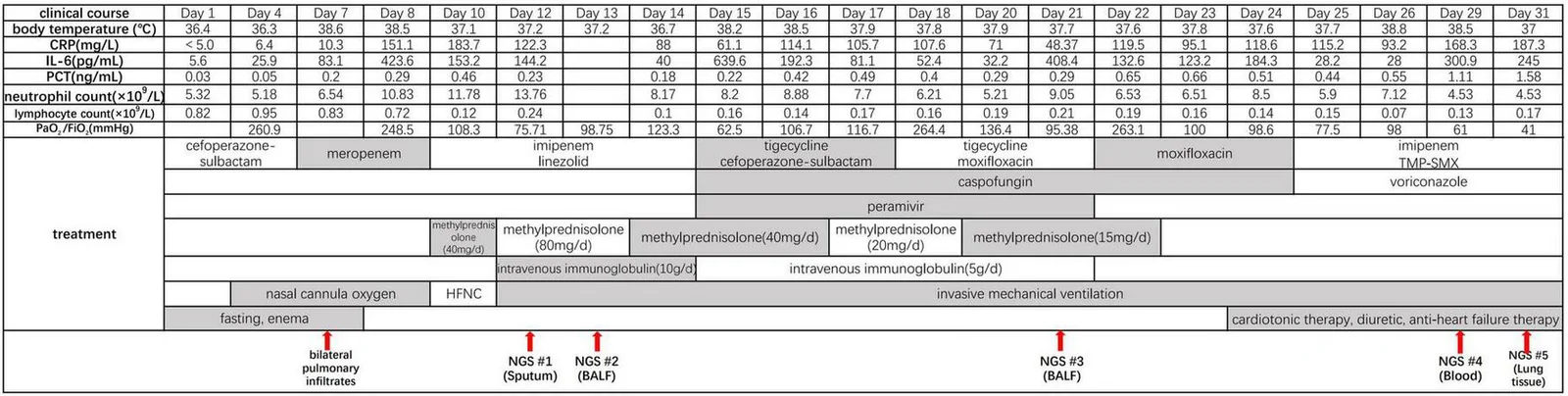
Timeline of clinical course, laboratory parameters and therapeutic interventions throughout the entire hospitalization episode, from symptom onset (Day 1, 26 February 2024) to patient death (Day 31, 27 March 2024). Key clinical events, treatment regimens, and serial mNGS tests with corresponding specimen sources are labeled at matching time points. BALF, bronchoalveolar lavage fluid; mNGS, metagenomic next-generation sequencing; HFNC, high-flow nasal cannula.

**FIGURE 2:**
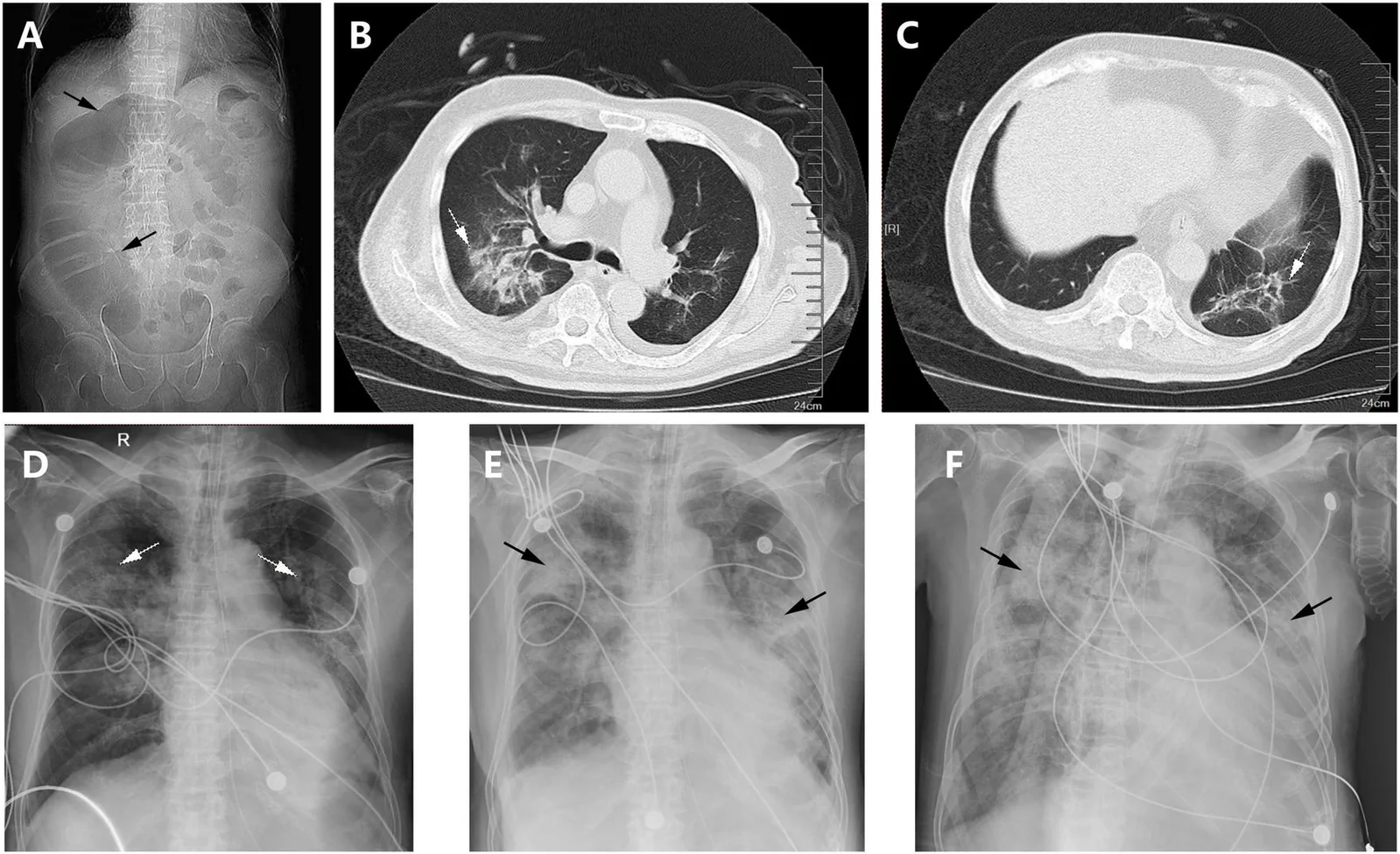
Chest and abdominal imaging. **(A)** Abdominal CT on Day 1 (26 February 2024). **(B,C)** Chest CT on Day 7 (two representative sections). **(D)** Bedside chest X-ray on Day 12. **(E**) Bedside chest X-ray on Day 23. **(F)** Bedside chest X-ray on Day 29. Arrows indicate representative lesions.

Fever persisted with progressive dyspnea and hypoxemia. On Day 10, while on 48% oxygen, arterial blood gas showed hypoxemia (PaO_2_ 52 mmHg). Tests for COVID-19, respiratory pathogens, and influenza were negative; sputum cultures grew only normal flora. Chest X-ray showed progressive bilateral infiltrates. After excluding cardiogenic pulmonary edema, the diagnosis of ARDS was established. Treatment was escalated to imipenem/cilastatin plus linezolid, and high-flow nasal cannula oxygen was initiated. On Day 11, methylprednisolone and intravenous immunoglobulin (IVIG) were added due to lack of improvement. On Day 12, his condition worsened with tachypnea, tachycardia, and SpO_2_ 88% on high-flow oxygen (FiO_2_ 70%). Endotracheal intubation and mechanical ventilation were performed. Chest X-ray showed enlarging bilateral opacities (Figure 2D). The first mNGS assay (sputum) identified no definite pathogens.

The detailed treatment regimen, including drug durations, and adjustments, is presented in the timeline figure (Figure 1). Despite continued antibiotics and corticosteroids, the patient deteriorated. On Day 13, the second mNGS (BALF) showed only low, non-replicating CMV. The antimicrobial regimen was adjusted over the following days to include tigecycline, caspofungin, peramivir, and moxifloxacin (Figure 1). On Day 21, the third mNGS (BALF) revealed predominant HSV-1 with high DNA/RNA reads, with concurrent detection of *Candida* and *Aspergillus* (Table 1). By the time HSV-1 was confirmed by the third BALF, the patient had already developed heart failure and gastrointestinal bleeding. Acyclovir was not administered due to the need for fluid restriction, the uncertain benefit of antiviral therapy, and greater clinical concern for fungal and bacterial co-infections. By Day 23, the bilateral opacities had become more confluent and extended to a wider lung area (Figure 2E).

On Day 24, the patient developed respiratory distress, peripheral edema, elevated BNP, and bilateral pleural effusion, requiring cardiotonic and diuretic support. On Day 25, after multidisciplinary consultation, therapy was changed to tigecycline, voriconazole, and trimethoprim-sulfamethoxazole (TMP-SMX). By Day 29, pulmonary infiltrates had progressed further (Figure 2F), the lymphocyte count remained extremely low (<0.20 × 10^9^/L), and oxygenation did not improve. On Day 31, SpO_2_ dropped to 59%, procalcitonin was 1.58 ng/mL, and blood gas showed severe hypercapnia and hypoxemia (PaCO_2_ 84 mmHg, PaO_2_ 41 mmHg). The fourth mNGS (blood) demonstrated active HSV-1 viremia (Table 1).

The patient subsequently developed multiple organ dysfunction and died despite aggressive resuscitation. After obtaining informed consent from the family, a percutaneous lung puncture biopsy was performed. Histopathology showed scattered inflammatory cell infiltration, well-differentiated alveolar epithelial cells, and a small amount of skin tissue (indicating limited sampling). The fifth mNGS assay (lung tissue) detected low-level Human polyomavirus 7 (HPyV7) with no other pathogens.

## Discussion

The close temporal relationship between intestinal obstruction and ARDS onset is consistent with a gut-lung axis mechanism. Intestinal obstruction can disrupt the gut epithelial barrier, leading to translocation of microbial products (e.g., lipopolysaccharide) without viable bacteria, triggering systemic inflammation—“sterile inflammation” (1, 12). This systemic inflammatory state, coupled with acute stress and age-related immunosenescence (13, 14), may have created a permissive environment for HSV-1 reactivation. Thus, the intestinal obstruction was likely the priming event for ARDS and subsequent HSV-1 reactivation. This proposed causal chain, while biologically plausible, remains speculative and requires further mechanistic studies.

Whether HSV-1 causes or contributes to increased morbidity or mortality in patients with lower respiratory tract infections, or whether it is merely an epiphenomenon in a more severely ill host, is still controversial (15). In this case, however, multiple lines of evidence suggest that HSV-1 played a significant role in the progressive deterioration of ARDS: (1) temporal sequence—absent in early specimens, massive in BALF on day 21, then in blood by day 29; (2) high read abundance and transcriptional activity—RNA reads were high in both BALF and blood (13,120 and 12,949, respectively), confirming active viral replication (3, 8) consistency across specimens—the same virus was identified in both BALF and blood; (4) biological plausibility—advanced age (91 years) and acute stress from intestinal obstruction facilitated HSV-1 reactivation (5, 13) exclusion of alternatives—no other pathogens were found at clinically significant levels. Collectively, this evidence argues against HSV-1 being merely an innocent bystander in this patient. Nevertheless, we acknowledge that without histopathological confirmation (e.g., immunohistochemistry or HSV-specific staining), causality cannot be definitively established. Therefore, we propose that HSV-1 reactivation likely played a significant contributing role in the progressive and fatal ARDS trajectory, while recognizing that the evidence remains circumstantial. The decision not to initiate acyclovir reflects a potential gap in clinical judgment and limited experience with antiviral management in this context.

Meanwhile, the patient presented with persistent leukocytosis (peak 12.0 × 10^9^/L) and elevated CRP (151.1 mg/L), findings that might raise concern for bacterial co-infection. However, severe viral pneumonia can itself induce marked leukocytosis via cytokine release. Elevated IL-6 (423.6 pg/mL) potently stimulates neutrophil release from the bone marrow (16). The profound lymphocytopenia (absolute lymphocyte count < 0.02 × 10^9^/L) further supports a viral etiology. The absence of a marked PCT elevation throughout most of the clinical course also argues against a clinically significant bacterial infection, as does the absence of any bacterial signal across five mNGS assays. Prior broad-spectrum antibiotic exposure may have contributed to the persistently negative conventional bacterial and fungal cultures. Studies have shown that with increasing antibiotic exposure time, culture positivity declines significantly, whereas mNGS positivity remains relatively stable (17). Prolonged antibiotic use (>7 days) markedly reduces culture positivity (from 86.3% to 54.8%), while the decline in mNGS sensitivity is smaller (from 92.2% to 76.2%) (18). Regardless of antibiotic exposure, the positivity rate of mNGS was significantly higher than that of culture (80.77% vs. 37.18%) (19). Importantly, despite repeated antibiotic adjustments, conventional pathogen testing from multiple sites and time points consistently yielded negative results, yet the patient’s clinical condition continued to deteriorate rapidly—a phenomenon rarely encountered in clinical practice. This strongly suggests the presence of pathogens that conventional methods are inherently unable to detect, underscoring the critical value of mNGS (20).

Metagenomic next-generation sequencing has emerged as a transformative tool in infectious disease diagnostics by enabling simultaneous, hypothesis-free detection of a broad array of pathogens directly from clinical specimens. The application of mNGS can significantly enhance the pathogen detection rate and optimize antimicrobial drug management in immunocompromised patients with lower respiratory tract infections (21). This technique bypasses the limitations of conventional tests, offering a major advantage in diagnosing infections caused by fastidious, unculturable, or rare pathogens (22). However, despite its promise, the translation of mNGS from a research tool to routine clinical practice is hampered by several persistent challenges. A primary difficulty lies in the interpretation of its comprehensive, yet often complex, results. On one hand, a negative result does not provide a definitive answer. mNGS can produce false negatives, especially for pathogens that are difficult to lyse, such as mycobacteria and certain fungi. In elderly individuals, HSV-1 can reactivate intermittently at low levels (23). Since mNGS detection is influenced by viral load, the initial negative results do not exclude the presence of low-level HSV-1. Conversely, the test’s high sensitivity can lead to the detection of incidental background noise or colonizers. On the other hand, discriminating infection from colonization is a formidable task (24). Specimen type substantially influences diagnostic yield, as sampling at the site of primary infection yields the highest diagnostic return (25). In this case, the negative sputum mNGS result likely reflected the limited sensitivity of sputum samples for lower respiratory tract pathogens compared to BALF, whereas the subsequent BALF and blood specimens provided more clinically relevant information. Sampling timing also critically affects detection, since pathogen burden fluctuates over the disease course; samples obtained after prolonged empirical antimicrobial treatment may have reduced sensitivity, and the timing of collection can substantially influence microbiome analyses. Variability in sequencing workflows—including differences in library preparation, sequencing depth, bioinformatic pipelines, and reference databases—can further affect quantitative read counts and reproducibility across laboratories (26, 27).

To navigate this challenge, clinical decision-making is aided by a multi-dimensional approach that integrates multiple factors. Although no universal standard exists, incorporating clinical context (host factors), specimen quality, the abundance of sequencing reads, the relative abundance of species, and the use of RNA sequencing (RNA-mNGS) to detect transcriptional activity have all been shown to improve interpretive accuracy. RNA-mNGS, in particular, can offer a distinct advantage as its detection of RNA reflects active replication, distinguishing a viable pathogen from a dormant colonizer or contaminant (28). Collectively, these technical factors collectively indicate that mNGS read numbers should not be interpreted as absolute quantitative measures but rather as semiquantitative signals that must be integrated with clinical context, temporal dynamics, and multi-specimen consistency. In this context, the longitudinal trend within the same patient is likely to be more informative than the absolute read count from a single specimen.

Recent advances in background genomic filters have further improved the ability to differentiate microbial signals from environmental or procedural contamination. The BECLEAN model, which applies a library concentration-normalized approach to distinguish true pathogens from background noise, has demonstrated a diagnostic accuracy of 92.9% in clinical mNGS interpretation (29). Hospital-specific background microbial libraries have also been proposed as a complementary strategy to reduce false positives and enhance diagnostic accuracy (30). In parallel, advances in host DNA depletion techniques have further improved microbial signal detection. Nucleosome-targeted host DNA depletion can reduce human DNA background by an average of 66-fold, consequently enriching microbial reads by approximately 46.73-fold (31). Host DNA depletion-assisted mNGS has been shown to significantly improve diagnostic sensitivity in clinical specimens such as bronchoalveolar lavage fluid (32). These technical and bioinformatic advances collectively provide a framework for reducing diagnostic subjectivity in mNGS interpretation. While this reduces environmental and procedural noise, low-abundance true pathogens may still be overlooked, and clinical correlation remains essential for final interpretation.

The initial sputum mNGS was confounded by oral contamination, a well-known limitation of sputum samples. The first BALF mNGS was performed early, possibly before HSV-1 had reactivated to detectable levels. CMV detection in the lower respiratory tract of critically ill patients should be interpreted with caution, as it often represents latency or shedding rather than true pneumonitis (33, 34). The second mNGS assay detected CMV DNA at very low reads (eight reads) with zero RNA activity, providing no evidence of active replication. These findings are consistent with latent reactivation without clinical significance, a scenario frequently encountered in critically ill elderly patients. mNGS analysis of the second BALF specimen detected *Mycoplasma salivarium* with 355 RNA reads. The species is a known oropharyngeal commensal and was labeled as a suspected background microorganism by the sequencing platform. The comparatively high RNA read count brings interpretive uncertainty, given that sampling contaminants usually show low sequencing signals. However, clinical evidence does not support its pathogenic role. Fluoroquinolones, which have in vitro activity against this *mycoplasma*, failed to reverse respiratory deterioration. Moreover, it was detected only on a single occasion without temporal consistency with worsening hypoxemia. Therefore, this finding was attributed to oropharyngeal contamination during bronchoscopy. In the fifth assay, HPyV7 was detected at a very low level (36 DNA reads). HPyV7 is primarily associated with pruritic dermatosis in immunocompromised patients (35); its role as a respiratory pathogen is extremely limited. The low read count, absence of cutaneous lesions, and lack of detection in prior specimens argue against pathogenicity. In the third assay, *Candida parapsilosis* (high RNA) and *Aspergillus flavus* (moderate reads) were also detected. Fungal cultures from multiple specimen types yielded only low-level *Candida* growth (1+) on two occasions, all susceptible to fluconazole and voriconazole, but the patient showed no clinical response to antifungals. All four GM tests were negative; the two mildly elevated G tests occurred during IVIG and albumin infusions, both known to cause false-positive results. BALF *Aspergillus* detection was taken seriously, and antifungal therapy was adjusted from caspofungin to voriconazole, but without clinical improvement. Blood mNGS detected no fungal sequences, and no radiographic features of invasive fungal disease were present. Given that *Candida* pneumonia is exceptionally rare in non-neutropenic patients (36), and repeated sputum fungal cultures, serum G-test, and GM-test were all negative, these findings are most consistent with colonization rather than invasive infection (37). We acknowledge the possibility of fungal colonization or low-grade infection, but the available evidence does not support it as the primary driver of fatal ARDS. The clinical trajectory of this case underscores a recurring diagnostic challenge in elderly patients with unexplained ARDS. Serial mNGS can provide a dynamic view of pathogen emergence that single-time-point assays cannot offer.

## Conclusion

Several limitations should be acknowledged. First, autopsy was not performed, and the percutaneous postmortem lung biopsy yielded only a small tissue specimen, precluding immunohistochemistry or HSV-specific staining and thereby limiting the strength of causal inferences. As a single case report, our findings may not be generalizable. In addition, patient-reported outcomes could not be obtained, as the patient died during hospitalization. Nevertheless, this case suggests that incomplete intestinal obstruction may serve as a trigger for ARDS in elderly patients, and that pulmonary HSV-1 reactivation may contribute to progressive lung injury and fatal ARDS, even in the absence of typical herpetic skin lesions. For patients with negative conventional testing, mNGS expands the diagnostic spectrum, and serial monitoring underscores the diagnostic value of longitudinal testing in elderly patients with rapidly progressive ARDS of unclear etiology. Accurate interpretation of mNGS findings, however, requires careful integration of clinical context, read abundance, RNA activity, and consistency across multiple specimens.

## Data Availability

The original contributions presented in this study are included in this article/supplementary material, further inquiries can be directed to the corresponding author.
